# A novel P20R mutation in the alpha-B crystallin gene causes autosomal dominant congenital posterior polar cataracts in a Chinese family

**DOI:** 10.1186/1471-2415-14-108

**Published:** 2014-09-08

**Authors:** Xin-Yi Xia, Qiu-Yue Wu, Li-Mei An, Wei-Wei Li, Na Li, Tian-Fu Li, Cui Zhang, Ying-Xia Cui, Xiao-Jun Li, Chun-Yan Xue

**Affiliations:** Institute of Laboratory Medicine, Jinling Hospital, Nanjing University School of Medicine, 305 East Zhongshan Road, Nanjing, 210002 People’s Republic of China; Department of Ophthalmology, Jinling Hospital, Nanjing University School of Medicine, Nanjing, People’s Republic of China

**Keywords:** Congenital posterior polar cataract, Alpha-B crystallin gene, P20R mutation

## Abstract

**Background:**

To identify the genetic defects and investigate the possible mechanism of cataract genesis in a five-generation family with autosomal dominant congenital posterior polar cataracts.

**Methods:**

Clinical data were collected, and the lens phenotypes of the affected members in this family were recorded by slit lamp photography. Genomic DNA was isolated from peripheral blood using QIAamp DNA Blood Mini Kits. Twenty-three mutational hot spots associated with autosomal dominant congenital posterior polar cataracts were screened by PCR-based DNA sequencing. Properties and structural models of wild-type and mutant alpha-B (αB)-crystallin (CRYAB) were generated and analyzed using SWISS-MODEL.

**Results:**

All affected individuals in this family started to exhibit poor vision at the age of 8–10 years. The lens opacity consisted of a single, well-defined plaque, 0.5–3 mm in diameter, which was confined to the posterior pole of the lens. DNA sequencing analysis of the affected members showed a novel, heterozygous missense mutation c.59C > G (P20R) in exon 1 of the *CRYAB* gene. This mutation was not found in 10 unaffected family members, or in 200 unaffected and unrelated individuals, thereby excluding the possibility that it is a rare polymorphism. Data generated using the ProtScale and PyMOL programs revealed that the mutation altered the stability and solubility of the αB-crystallin protein.

**Conclusions:**

This study reported a novel c.59C > G (P20R) missense mutation in *CRYAB* in a five-generation Chinese family with posterior polar cataract.

**Electronic supplementary material:**

The online version of this article (doi:10.1186/1471-2415-14-108) contains supplementary material, which is available to authorized users.

## Background

Hereditary congenital cataract [OMIM 604307] is an opacification of the lens of the eye, which causes visual impairment or even blindness during infancy or early childhood. Cataracts can be clinically classified into many different types, such as posterior polar, anterior polar, and lamellar. Around one-third of congenital cataracts can be inherited in an autosomal dominant, autosomal recessive, or X-linked manner, although autosomal dominant inheritance of congenital cataracts predominates. Thus far, congenital cataracts have been associated with the following genes: 10 crystallin genes (*CRYAA, CRYAB, CRYBB1, CRYBB2, CRYBA1/A3, CRYGA, CRYGB, CRYGC, CRYGD, CRYGS*) [1–4], 3 membrane protein genes (*GJA3, GJA8* and *MIP*) [5–7], beaded filament structural protein-2 (*BFSP2*) [8], a ferritin light chain gene (*TFL*) [9], transcription regulatory factors genes (*MAF, PITX3, HSF4, PAX6*) [10–12], connexin 46 (*Cx46*) [13], aspartate-47 of the Connexin50 gene (*Cx50D47*) [14], a receptor tyrosine kinase gene (*EPHA2*) [15], and the Wolfram gene (*WFS1*) [16].

The water-soluble crystallins are the most abundant proteins in the lens and have a critical role in maintaining lens transparency. They are mainly composed of two related proteins, αA- and αB-crystallins, encoded by the *CRYAA* and *CRYAB* genes, which are present in a 3:1 ratio. Both proteins belong to the small heat shock protein (sHSP) family and function as molecular chaperones to prevent the stress-induced aggregation of other proteins [17]. αA- and αB-crystallins form hetero-oligomers that bind and sequester damaged proteins, preventing the formation of particulates that scatter light [18]. *CRYAA* is mainly expressed at a high level in the lens, while *CRYAB* is widely expressed in a variety of tissues and is associated with neurologic, cardiac, and muscular disorders [18]. The *CRYAB* gene comprises three exons and encodes a small, 175 amino acid protein belonging to the sHSP family [19], which acts as a molecular chaperone, preventing the aggregation of denatured proteins after the exposure to stresses, such as heat shock, radiation, oxidative stress and anticancer drugs [20]. Besides being found in the lens, αB-crystallin is distributed in other tissues and organs, including the brain, heart, stomach, lung, kidney, muscle, and retina. Mutations in the *CRYAB* gene cause distinct clinical phenotypes, including isolated cataract, myofibrillar myopathy, cardiomyopathy, or a multi-systemic disorder combining these features [18].

In the present study, we first investigated a five-generation Chinese family with autosomal dominant, isolated, congenital, posterior polar cataract and identified a novel missense mutation in exon 1 of *CRYAB* that leads to an exchange of proline for arginine at codon 20 (P20R).

## Methods

### Participant and clinical data

**Figure 1:**
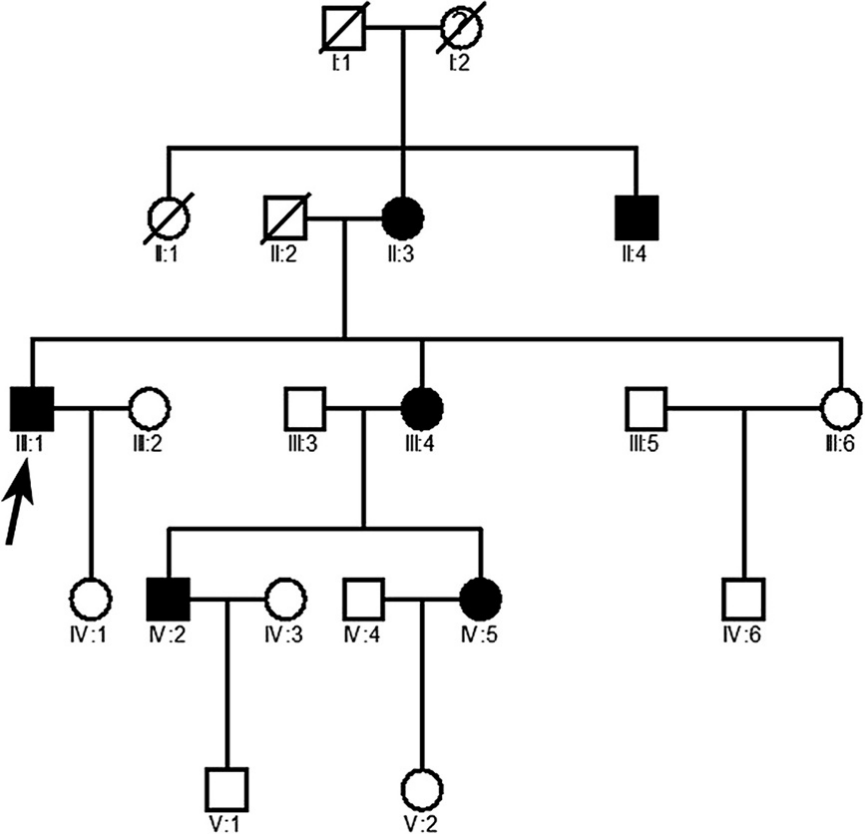
**The Chinese pedigree with congenital posterior polar cataract.** The transmission pattern suggests the cataract is inherited in an autosomal dominant manner. Square symbols: males; round symbols: females; shaded symbols: ophthalmologist-confirmed posterior polar cataract; question mark (I2 member in the family): a poor vision which was suspected to have a cataract, but could not be diagnosed clearly. arrow: proband.

A five-generation Chinese cataract family was enrolled at Nanjing General Hospital of the Nanjing Military Region Ophthalmic Center. Sixteen living family members (Figure 1), including 6 affected and 10 unaffected subjects, underwent full ophthalmic examinations, including visual acuity, slit-lamp microscopy, fundus examination, intraocular pressure, and B-ultrasonic scanning. Additionally, they underwent a systematic medical assessment, which included serum creatine kinase level, electrocardiography (ECG), echocardiography, muscular tension, and muscular reflexes of the proximal and distal muscles of the lower and upper limbs. Two hundred unrelated and unaffected individuals were collected to be normal controls in the study.

All procedures used in the study confirmed to the tenets of the Declaration of Helsinki. The Ethics Committee of Jinling Hospital approved all study protocols. All participants had knowledge of their participation in the study. Written informed consent was obtained from all participants.

### Mutation screening

Genomic DNA was extracted from the peripheral blood of the patients using QIAamp DNA Blood Kits (Qiagen, German). Mutation screening was performed using a candidate gene approach. Known candidate genes for hereditary cataracts, such as *CRYAA, CRYAB, CRYBA1/3, CRYBB1, CRYBB2, CRYGA-D, CRYGS, GJA3, GJA8, MIP, HSF4, BFSP2, FTL, MAF, PITX3, PAX6, Cx46, Cx50D47, EPHA2* and *WFS1* were analyzed by polymerase chain reaction (PCR) amplification, followed by direct DNA sequencing. The specific primer pairs are given in Additional file 1. The sequencing results were analyzed using Chromas (version 2.3) and compared with reference sequences in the National Center for Biotechnology Information (NCBI) database.

### Bioinformatics analysis of protein structures and properties

Biophysical predictions of the altered αB-crystallin protein were analyzed using bioinformatics tools. In particular, we used ProtScale (provided by the Swiss Institute of Bioinformatics, Geneva, Switzerland) to examine hydrophilicity. The resulting protein database files were visualized using Swiss-PDB Viewer (version 4.01, provided by the Swiss Institute of Bioinformatics, Geneva, Switzerland).

### Alignment analysis

ClustalW (version 1.83) was used to compare the sequence of *CRYAB* (*Homo sapiens*, NP_001276736.1) with orthologs of *Rattus norvegicus* (NP_037067.1), *Danio rerio* (NP_571232.1), *Ovis aries* (NP_001012475.1), *Sus scrofa* (XP_005667376.1), and *Pongo abelii* (NP_001125917.1).

## Results

### Clinical findings

**Figure 2:**
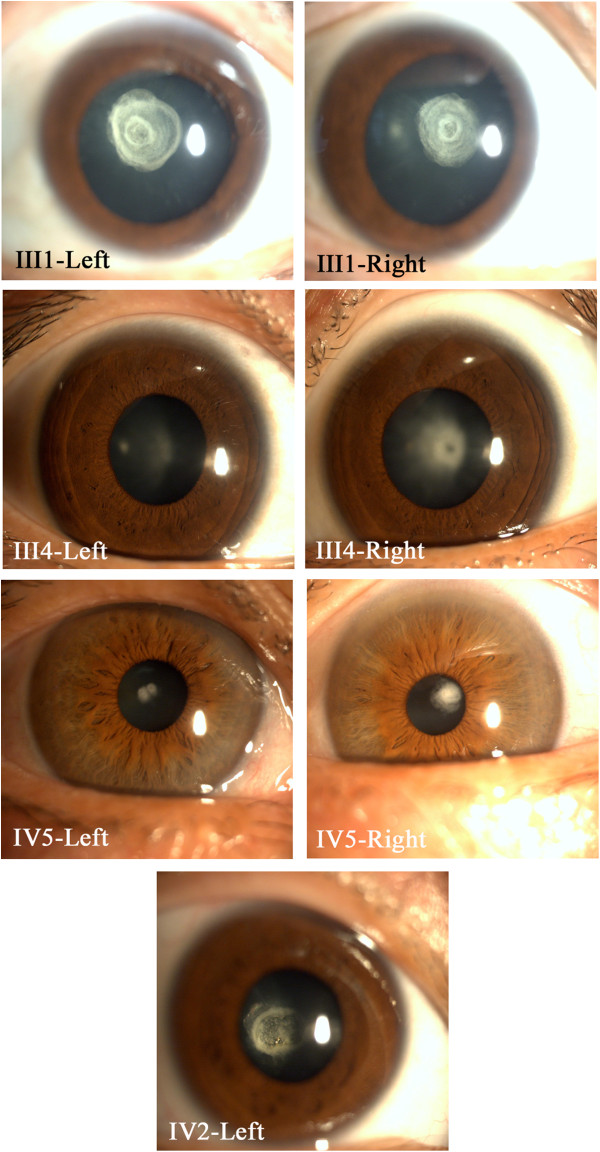
**The part of slit-lamp photograph of the dilated eye of the affected family member (III1, III4, IV2 and IV5).** The opacity consisted of a single well-defined plaque, 0.5–3 mm in diameter, which was confined to the posterior pole of the lens. The slit lamp pictures of II3 and II4 could not be got because of surgery in the other hospital. The picture of Oculus Dexter in IV2 had a bad quality that could not be offered.

The cataracts in this family were inherited in an autosomal dominant manner and appeared to be of the congenital, posterior polar type. All affected individuals started to have poor vision at the age of 8–10 years. Lens opacity, which was bilateral in all cases, consisted of a single, well-defined plaque, 0.5–3 mm in diameter, which was confined to the posterior pole of the lens (Figure 2). Clinical investigation including visual acuity, age of onset in each affected subjects were described in Table 1. No other systemic findings in the cardiovascular and muscular systems were identified in any of the affected members.

**Table 1:** **Clinical data in each affected subjects**

Number	Onset Age	Op. Age	Preop. BCVA		Postop. BCVA	
			OD	OS	OD	OS
II3	No available	16	No available	No available	20/50	20/40
II4	No available	17	No available	No available	20/40	20/30
III1	12	20	20/200	20/100	20/40	20/30
III4	10	19	20/100	20/125	20/30	20/50
IV2	9	11	20/100	20/80	20/25	20/25
IV5	8	No available	20/40	20/50	No available	No available

Op.: operation; BCVA: best corrected visual acuity; OD: Oculus Dexter; OS: Oculus Sinister

### Sequence analysis

**Figure 3:**
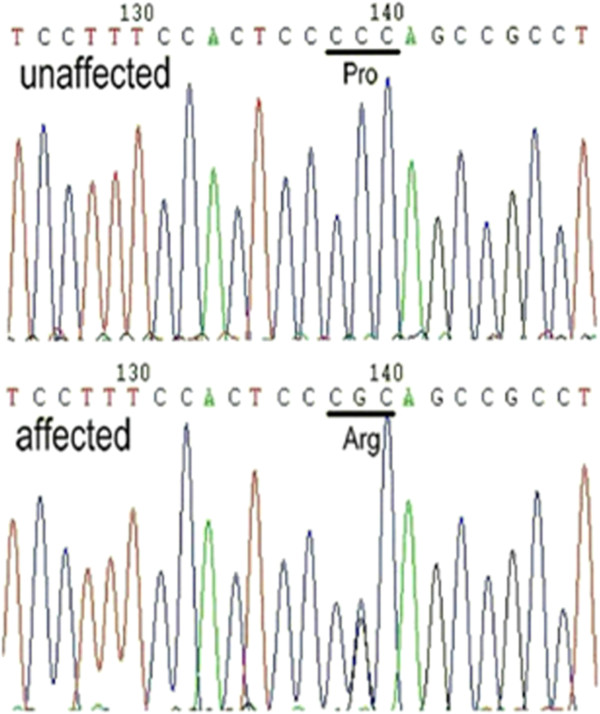
**Sequence analysis of exon 1 of the** ***CRYAB*** **gene.** All six affected members carried a novel C-G heterozygous mutation at nucleotide 59 in exon 1 of the *CRYAB* gene, which led to an exchange of proline for arginine at codon 20 (P20R); this mutation was not found in 10 unaffected family members.

**Figure 4:**
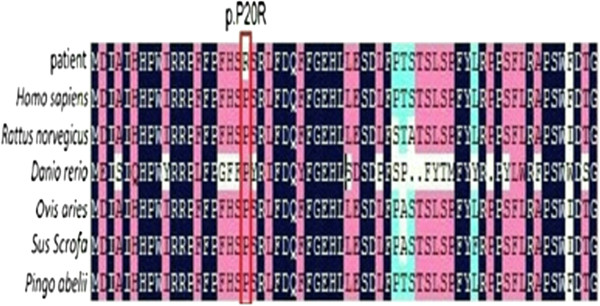
**Protein sequence alignments.** The P20R substitution of the alpha-B crystallin polypeptide occurs in a highly conserved amino acid residue, as shown in this cross-species alignment.

Multiple genes that could potentially cause congenital cataracts were screened by PCR-based DNA sequencing. No mutation was found in any gene, except for *CRYAB*. Sequencing analysis revealed that the *CRYAB* gene segment carried a novel C > G heterozygous mutation at nucleotide 59 in exon 1 of the gene in all six affected family members. This mutation was not found in the 10 unaffected members or in the 200 unaffected and unrelated normal controls (Figure 3). The c.59C > G (P20R) mutation in *CRYAB* was not listed in the NCBI SNP database (dbSNP), Exome Variant Server or Human Gene Mutation Database (HGMD), which excludes the possibility that it is a rare polymorphism. At the protein level, it led to an amino acid change from proline to arginine at amino acid 20 (P20R). This indicated that the missense mutation (c.59C > G) was the cause of posterior polar cataract in this pedigree. In addition, the Pro20 residue is highly conserved across species, as shown in Figure 4.

### Bioinformatics analysis

**Figure 5:**
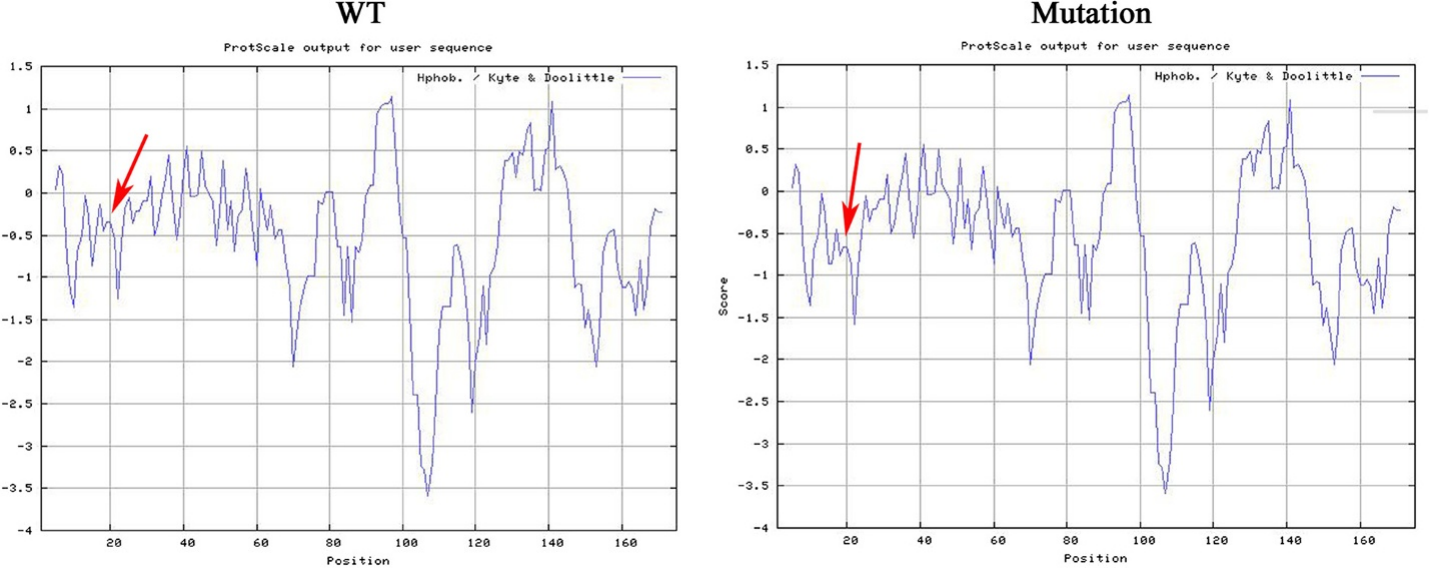
**Hydropathy plot of wild-type and mutated αB-crystallin.** Obviously, the mutant αB-crystallin showed lower hydrophilicity in the mutated region compared with the wild-type protein (indicated by red arrows).

To better understand the effects of the mutation on the biochemical properties and structure of the αB-crystallin protein, the hydrophilicity of the corresponding region was predicted using the program ProtScale, which indicated that the mutant αB-crystallin showed lower hydrophilicity in the mutated region compared with the wild-type protein (Figure 5). Consistent with the change of hydrophilicity, the isoelectric point (pI) of the αB-crystallin protein also changed, from pH 6.90 in the wild-type protein to pH 8.56 in the mutant protein.

## Discussion

Posterior polar cataract is a significant opacity that is distributed at the back of the lens. Because of its proximity to the optical center of the eye, it can have a great effect on visual acuity [21]. Here, we reported a novel c.59C > G (P20R) missense mutation in the *CRYAB* gene in a five-generation Chinese family with posterior polar cataract. The alpha-B crystallin protein encoded by *CRYAB* is composed of three domains: an NH2-terminal tail, the αB crystallin domain (ACD), and a COOH-terminal extension. The NH2-terminal domain is encoded by the first exon, which controls the chaperone function of the protein [22]. The P20R mutation is located in the NH2-terminal domain, and the Pro20 residue is highly conserved across species, which suggests that it plays a key role in the structure and function of the protein.

Since the first description of a *CRYAB* mutation (R120G) leading to associated disease in 1998 [14], only a few additional mutations have been reported. These mutations cause markedly different clinical features, such as isolated cataract, myofibrillar myopathy, cardiomyopathy, or a multi-systemic disorder combining these phenotypes [13].

Thus far, eight mutations in the *CRYAB* gene associated with isolated cataract have been described, including this study. All eight *CRYAB* mutations and the corresponding clinical features are summarized in Table 2 [3, 18, 20, 21, 23–25]. Most reported *CRYAB* mutations (7/8) were dominant, although Safieh et al. reported that an autosomal recessive mutation, R56W, resulted in cataract [23]. Four out of the eight isolated cataract-causing mutations are located in exon 1. It is noted that two mutations occur at an evolutionarily conserved residue, Pro20, indicating a potential hotspot involved in cataract. It seems that mutations outside the α-crystallin domain result in isolated cataract. In 2006, Liu et al. stated that a family with isolated posterior polar cataract carried a P20S mutation [3]. As αB-crystallin interacts with αA-crystallin, a mutant αB-crystallin with the P20S mutation may act in a dominant negative manner, thereby interfering with the function of αA-crystallin and leading to the formation of cataracts early in the lives of individuals who carry the mutation. Additionally, the mutant αB-crystallin no longer possessed the ability to inhibit apoptosis of lens epithelial cells, which resulted in the development of cataracts. The paper reported by Li et al. also confirmed that the cataract causing P20S mutation of αB-crystallin impaired the chaperone activity of αA-crystallin and induced apoptosis of human lens epithelial cells [22]. In a recent study, it was suggested that the P20S mutation altered the secondary structure of the αB-crystallin protein, as shown by increasing α-helical and decreasing β-sheet contents, and changed the tertiary structure, which may influence protein stability and conformation and induce the formation of aggregates [26]. Our results indicated that P20R mutation changes the hydrophilicity and isoelectric point, but not the secondary and tertiary structure, of αB-crystallin, potentially lowering the solubility of the mutant protein. Therefore, we speculate that the P20R mutation lowers the solubility of the mutant αB-crystallin, causing protein self-aggregation and resulting in a more stable heteroaggregate with αA-crystallin. Additionally, it may affect the chaperone function of α-crystallin, thereby further contributing to the development of cataracts.

**Table 2:** ***CRYAB*** **gene mutations associated with isolated congenital cataracts**

No.	Inheritance mode	Exon	Nucleotide change	Amino acid change	Clinical features	Reference
1	AD	1	c.32 G > A	p.R11H	Nuclear cataract	[18]
2	AD	1	c.58 C > T	p.P20S	Posterior polar cataract	[3]
3	AD	1	c.59 C > G	p.P20R	Posterior polar cataract	Present study
4	AR	1	c.166 C > T	p.R56W	Dense complete white cataracts	[23]
5	AD	2	c.205 C > T	p.R69C	N/A	[24]
6	AD	3	c.418 G > A	p.D140N	Lamellar cataract	[25]
7	AD	3	c.450 del A	Frameshift	Posterior polar cataract	[20]
8	AD	3	c.557 G > A	p.A171T	Lamellar cataract	[21]

AD, Autosomal dominant; AR, Autosomal recessive; N/A, not available.

## Conclusions

Here, we reported a novel c.59C > G (P20R) missense mutation in *CRYAB* in a five-generation Chinese family with posterior polar cataract. Additionally, the P20R mutation changed the hydrophilicity and isoelectric point of αB-crystallin to lower the solubility of the mutant protein.

## Electronic supplementary material

Additional file 1: **Table Primers for mutational analysis of congenital cataract.** (DOC 184 KB)

## Abbreviations

CRYAA: Crystallin, alpha A
CRYAB: Crystallin, alpha B
CRYBB1: Crystallin, beta B1
CRYBB2: Crystallin, beta B2
CRYBA1: Crystallin, beta A1
CRYBA2: Crystallin, beta A2
CRYBA3: Crystallin, betaA3
CRYGA: Crystallin, gamma A
CRYGB: Crystallin, gamma B
CRYGC: Crystallin, gamma C
CRYGD: Crystallin, gamma D
CRYGS: Crystallin, gamma S
GJA3: Gap junction protein, alpha 3
GJA8: Gap junction protein, alpha 8
MIP: Major intrinsic protein of lens fiber
BFSP2: Beaded filament structural protein-2
FTL: Ferritin light chain gene
MAF: v-maf avian musculoaponeurotic fibrosarcoma oncogene homolog
PITX3: Paired-like homeodomain 3
HSF4: Heat shock transcription factor 4
PAX6: Paired box 6
Cx46: Connexin 46
Cx50D47: Geneaspartate-47 of Connexin50 gene
EPHA2: Receptor tyrosine kinase gene
WFS1: Wolfram gene
sHSP: Small heat shock proteins family
PCR: Polymerase Chain Reaction
pI: Isoelectric point
ACD: The αB crystallin domain
HGMD: Human Gene Mutation Database.

## References

[1] Lu S, Zhao C, Jiao H, Kere J, Tang X, Zhao F, Zhang X, Zhao K, Larsson C (2007). Two Chinese families with pulverulent congenital cataracts and deltaG91 CRYBA1 mutations. Mol Vis.

[2] Wang J, Ma X, Gu F, Liu NP, Hao XL, Wang KJ, Wang NL, Zhu SQ (2007). A missense mutation S228P in the CRYBB1 gene causes autosomal dominant congenital cataract. Chin Med J (Engl).

[3] Liu M, Ke T, Wang Z, Yang Q, Chang W, Jiang F, Tang Z, Li H, Ren X, Wang X, Wang T, Li Q, Yang J, Liu J, Wang QK (2006). Identification of a CRYAB mutation associated with autosomal dominant posterior polar cataract in a Chinese family. Invest Ophthalmol Vis Sci.

[4] Gu F, Li R, Ma XX, Shi LS, Huang SZ, Ma X (2006). A missense mutation in the gammaD-crystallin gene CRYGD associated with autosomal dominant congenital cataract in a Chinese family. Mol Vis.

[5] Guleria K, Sperling K, Singh D, Varon R, Singh JR, Vanita V (2007). A novel mutation in the connexin 46 (GJA3) gene associated with autosomal dominant congenital cataract in an Indian family. Mol Vis.

[6] Vanita V, Hennies HC, Singh D, Nurnberg P, Sperling K, Singh JR (2006). A novel mutation in GJA8 associated with autosomal dominant congenital cataract in a family of Indian origin. Mol Vis.

[7] Gu F, Zhai H, Li D, Zhao L, Li C, Huang S, Ma X (2007). A novel mutation in major intrinsic protein of the lens gene (MIP) underlies autosomal dominant cataract in a Chinese family. Mol Vis.

[8] Zhang L, Gao L, Li Z, Qin W, Gao W, Cui X, Feng G, Fu S, He L, Liu P (2006). Progressive sutural cataract associated with a BFSP2 mutation in a Chinese family. Mol Vis.

[9] Vanita V, Hejtmancik JF, Hennies HC, Guleria K, Nürnberg P, Singh D, Sperling K, Singh JR (2006). Sutural cataract associated with a mutation in the ferritin light chain gene (FTL) in a family of Indian origin. Mol Vis.

[10] Bu L, Jin Y, Shi Y, Chu R, Ban A, Eiberg H, Andres L, Jiang H, Zheng G, Qian M, Cui B, Xia Y, Liu J, Hu L, Zhao G, Hayden MR, Kong X (2002). Mutant DNA-binding domain of HSF4 is associated with autosomal dominant lamellar and Marner cataract. Nat Genet.

[11] Ke T, Wang QK, Ji B, Wang X, Liu P, Zhang X, Tang Z, Ren X, Liu M (2006). Novel HSF4 Mutation Causes Congenital Total White Cataract in a Chinese Family. Am J Ophthalmol.

[12] Burdon KP, McKay JD, Wirth MG, Russell-Eggit IM, Bhatti S, Ruddle JB, Dimasi D, Mackey DA, Craig JE (2006). The PITX3 gene in posterior polar congenital cataract in Australia. Mol Vis.

[13] Wang KJ, Zhu SQ (2012). A novel p. F206I mutation in Cx46 associated with autosomal dominant congenital cataract. Mol Vis.

[14] Berthoud VM, Minogue PJ, Yu H, Schroeder R, Snabb JI, Beyer EC (2013). Connexin50D47A decreases levels of fiber cell connexins and impairs lens fiber cell differentiation. Invest Ophthalmol Visual Sci.

[15] Dave A, Laurie K, Staffieri SE, Taranath D, Mackey DA, Mitchell P, Wang JJ, Craig JE, Burdon KP, Sharma S (2013). Mutations in the EPHA2 Gene Are a Major Contributor to Inherited Cataracts in South-Eastern Australia. PLoS One.

[16] Berry V, Gregory-Evans C, Emmett W, Waseem N, Raby J, Prescott D, Moore AT, Bhattacharya SS (2013). Wolfram gene (WFS1) mutation causes autosomal dominant congenital nuclear cataract in humans. Eur J Hum Genet.

[17] Numoto N, Kita A, Fujii N, Miki K (2012). A P39R mutation at the N-terminal domain of human αB-crystallin regulates its oligomeric state and chaperone-like activity. Biochem Biophys Res Commun.

[18] Chen Q, Ma J, Yan M, Mothobi ME, Liu Y, Zheng F (2009). A novel mutation in CRYAB associated with autosomal dominant congenital nuclear cataract in a Chinese family. Mol Vis.

[19] Sacconi S, Féasson L, Antoine JC, Pécheux C, Bernard R, Cobo AM, Casarin A, Salviati L, Desnuelle C, Urtizberea A (2012). A novel CRYAB mutation resulting in multisystemic disease. Neuromuscul Disord.

[20] Berry V, Francis P, Reddy MA, Collyer D, Vithana E, MacKay I, Dawson G, Carey AH, Moore A, Bhattacharya SS, Quinlan RA (2001). Alpha-B Crystallin Gene (CRYAB) Mutation Causes Dominant Congenital Posterior Polar Cataract in Humans. Am J Hum Genet.

[21] Devi RR, Yao W, Vijayalakshmi P, Sergeev YV, Sundaresan P, Hejtmancik JF (2008). Crystallin gene mutations in Indian families with inherited pediatric cataract. Mol Vis.

[22] Li H, Li C, Lu Q, Su T, Ke T, Li DW-C, Yuan M, Liu J, Ren X, Zhang Z, Zeng S, Wang QK, Liu M (2008). Cataract mutation P20S of αB-crystallin impairs chaperone activity of αA-crystallin and induces apoptosis of human lens epithelial cells. Biochim Biophys Acta.

[23] Safieh LA, Khan AO, Alkuraya FS (2009). Identification of a novel CRYAB mutation associated with autosomal recessive juvenile cataract in a Saudi family. Mor Vis.

[24] Sun W, Xiao X, Li S, Guo X, Zhang Q (2011). Mutation analysis of 12 genes in Chinese families with congenital cataracts. Mol Vis.

[25] Liu Y, Zhang X, Luo L, Wu M, Zeng R, Cheng G, Hu B, Liu B, Liang JJ, Shang F (2006). A novel αB-crystallin mutation associated with autosomal dominant congenital lamellar cataract. Invest Ophthalmol Vis Sci.

[26] Raju I, Abraham EC (2013). Mutants of human αB-crystallin cause enhanced protein aggregation and apoptosis in mammalian cells: Influence of co-expression of HspB1. Biochem Biophys Res Commun.

[CR27] The pre-publication history for this paper can be accessed here:http://www.biomedcentral.com/1471-2415/14/108/prepub

