# Supplementary material for: A novel P20R mutation in the alpha-B crystallin gene causes autosomal dominant congenital posterior polar cataracts in a Chinese family
Source: BMC Ophthalmol. 2014 Sep 8;14:108. doi: 10.1186/1471-2415-14-108 (PMC4169818; doi:10.1186/1471-2415-14-108)
Supplement: Supplementary file 1 — Additional file 1: Table Primers for mutational analysis of congenital cataract. (DOC 184 KB) [file 12886_2014_473_MOESM1_ESM.doc]

**Additional file 1. Table Primers for mutational analysis of congenital cataract.**

| **Primer** | **Exon** | **Primer sequence 5’-3’** | **Product size (bp)** |
| --- | --- | --- | --- |
| *CRYAA-1F* | 1 | CTCCAGGTCCCCGTGGTACCA | 254 |
| *CRYAA-1R* |  | GCGAGGAGAGGCCAGCACCAC |  |
| *CRYAA-2F* | 2 | CTGTCTCTGCCAACCCCAGCAG | 223 |
| *CRYAA-2R* |  | CCCCTGTCCCACCTCTCAGTGCC |  |
| *CRYAA-3F* | 3 | GGCAGCTTCTCTGGCATGGGG | 312 |
| *CRYAA-3R* |  | GGGGAGCCAGCCGAGGCAATG |  |
| *CRYAB 1F* | 1 | TGCATATATAAGGGGCTGGCTGTA | 363 |
| *CRYAB 1R* |  | CAGGGTAGGAAAGGAAAATGGATG |  |
| *CRYAB 2F* | 2 | AGGATGAATTACCCGGACAGAAAG | 360 |
| *CRYAB 2R* |  | ACCCCTGATCCCGACTGTTAT |  |
| *CRYAB 3F* | 3 | TGAGTTCTGGGCAGGTGATAATAGTT | 391 |
| *CRYAB 3R* |  | AGCTTGATAATTTGGGCCTGCC |  |
| *CRYBB1-2F* | 2 | TAGCGGGGTAATGGAGGGTG | 664 |
| *CRYBB1-2R* |  | AGGATAAGAGTCTGGGGAGGTGG |  |
| *CRYBB1-3F* | 3 | CCTGCACTGCTGGCTTTTATTTA | 475 |
| *CRYBB1-3R* |  | *TCTCCAGAGCCCAGAACCATG* |  |
| *CRYBB1-4F* | 4 | CCAACTCCAAGGAAACAGGCATA | 491 |
| *CRYBB1-4*R |  | CCTCCCTACCCACCATCATCTC |  |
| *CRYBB1-5F* | 5 | TAGACAGCAGTGGTCCCTGGAGA | 416 |
| *CRYBB1-5*R |  | AGCACTGGGAGACTGTGGAAGG |  |
| *CRYBB1-6*R | 6 | CCTAGAAAAGGAAACCGAGGCC | 551 |
| *CRYBB1-6*R |  | AGCGAGGAAGTCACATCCCAGTA |  |
| *CRYBB2-1F* | *1* | TCTGTGGGCATTTGCTGACCC | 292 |
| *CRYBB2-1R* |  | GCTAACAGCATTGAAGTCTCTGCCC |  |
| *CRYBB2-2F* | *2* | GACCCCACAGCTCTGGGACAGTC | 393 |
| *CRYBB2-2R* |  | GGAGGGACTTTCAGTATCAGCTCCAAC |  |
| *CRYBB2-3F* | 3 | CACGGCTGCTTATAGCCAGAGCC | 449 |
| *CRYBB2-3R* |  | TCTATCTGACTGCAAAGCATGAATTATCTCC |  |
| *CRYBB2-4F* | 4 | GCTTTGGGCACAGCGATGTTCTG | 744 |
| *CRYBB2-4R* |  | GGCCCCTTCCTGGTCCCCA |  |
| *CRYBB2-5F* | 5 | AGTGGTCATAGACACGTAGTGGGTGCAC | 706 |
| *CRYBB2-5R* |  | CTGTTCCCAAACTTAGGGACACACGC |  |
| *CRYBB2-6F* | 6 | CCCCTCGTTCACCCTCCCATCA | 506 |
| *CRYBB2-6R* |  | CACTGTGTCCAAGGTCACACAGCTAAGC |  |
| *CRYGA-2F* | 1&2 | AGGTCCCTTTTGTGTTGTTTTTGCC | 462 |
| *CRYGA-2R* |  | CATGAGGAATTATACGGCAGGATTGG |  |
| *CRYGA-3F* | 3 | CAGACCAGCTCGCACAAGTTAAGG | 353 |
| *CRYGA-3R* |  | AAGAGCCACTTAGTGCAGGGAACACAAC |  |
| *CRYGB-2F* | 1&2 | TGCAAATCCCCTACTCACCAAAATGG | 518 |
| *CRYGB-2R* |  | AAAAAGATGGAAGGCAAAGACAGAGCC |  |
| *CRYGB-3F* | 3 | TTTGTTTACTCTTGCGTTTTCTGTCTGCC | 410 |
| *CRYGB-3R* |  | GAAAGAAAGACAGGGCTCTACTAGTGCC |  |
| *CRYGC-2F* | 1&2 | TGCATAAAATCCCCTTACCGCTGAG | 522 |
| *CRYGC-2R* |  | ACTCTGGCGGCATGATGGAAATC |  |
| *CRYGC-3F* | 3 | AGACTCATTTGCTTTTTTCCATCCTTCTTTC | 407 |
| *CRYGC-3R* |  | GAAAGAATGACAGAAGTCAGCAATTGCC |  |
| *CRYGD-2F* | 1&2 | GCAGCCCCACCCGCTCA | 599 |
| *CRYGD-2R* |  | GGGTAATACTTTGCTTATGTGGGGAG |  |
| *CRYGD-3F* | 3 | TGCTTTTCTTCTCTTTTTATTTCTGGGTCC | 400 |
| *CRYGD-3R* |  | AGTAAAGAAAGACACAAGCAAATCAGTGCC |  |
| *CRYGS-1F* | 1 | ACTGAAACCAGCCCATAT | 209 |
| *CRYGS-1R* |  | CCTCTAGGCAAAGAAGCA |  |
| *CRYGS-2F* | 2 | CTTCATGTTCAGCCTTCA | 583 |
| *CRYGS-2R* |  | CCTCAGCAGCCAACAAGC |  |
| *CRYGS-3F* | 3 | CTTGACCTGCTGGTGATT | 405 |
| *CRYGS-3R* |  | TGATGATGCCTATTTGGA |  |
| *GJA8-L1F* | 1 | CGGGGCCTTCTTTGTTCTCTAGTCC | 877 |
| *GJA8-L1R* |  | AGGCCCAGGTGGCTCAACTCC |  |
| *GJA8-L2F* | 1 | CAGCCGGTGGCCCTGCC | 907 |
| *GJA8-L2R* |  | GTTGCCTGGAGTGCACTGCCC |  |
| *GJA3-1aF* | 1 | CTGCGATGCCTGTCCTGTGG 539 | 539 |
| *GJA3-1aR* |  | TTGTCCTGCGGTGGCTCCTT |  |
| *GJA3-1bF* | 1 | CGCCCACCCTCATCTACCT 549 | 549 |
| *GJA3-1bR* |  | GTGGGAACCCGATGGCAAC |  |
| *GJA3-1cF* | 1 | AGCTCAAGCAGGGCGTGACC 542 | 542 |
| *GJA3-1cR* |  | CAAGGGCGGCTGGTGCATCT |  |
| *GJA3-1dF* | 1 | CCCCGGCGCTCAAGGCTTAC 545 | 545 |
| *GJA3-1dR* |  | AACCCTTGTCCCCGCCACCC |  |
| *PAX6-1F* | 1 | CTCATTTCCCGCTCTGGTTC | 300 |
| *PAX6-1R* |  | AAGAGTGTGGGTGAGGAAGT |  |
| *PAX6-2F* | 2 | CACACTCTTTATCTCTCACTCTCCAGCC | 300 |
| *PAX6-2R* |  | AATAAAGCGAGAAAGAAGCGGAC |  |
| *PAX6-3F* | 3 | TCAGAGAGCCCATCGACGTAT | 300 |
| *PAX6-3R* |  | CTGTTTGTGGGTTTTGAGCC |  |
| *PAX6-4F* | 4 | TTGGGAGTTCAGGCCTACCT | 153 |
| *PAX6-4R* |  | GAAGTCCCAGAAAGACCAGA |  |
| *PAX6-5F* | 5 | CCTCTTCACTCTGCTCTCTT | 257 |
| *PAX6-5R* |  | ATGAAGAGAGGGCGTTGAGA |  |
| *PAX6-5aF* | 5a | TGAAAGTATCATCATATTTGTAG | 237 |
| *PAX6-5aR* |  | GGGAAGTGGACAGAAAACCA |  |
| *PAX6-6F* | 6 | GTGGTTTTCTGTCCACTTCC | 299 |
| *PAX6-6R* |  | AGGAGAGAGCATTGGGCTTA |  |
| *PAX6-7F* | 7 | CAGGAGACACTACCATTTGG | 252 |
| *PAX6-7R* |  | ATGCACATATGGAGAGCTGC |  |
| *PAX6-8F* | 8 | GGGAATGTTTTGGTGAGGCT | 371 |
| *PAX6-8R* |  | CAAAGGGCCCTGGCTAAATT |  |
| *PAX6-9F* | 9 | GTAGTTCTGGCACAATATGG | 206 |
| *PAX6-9R* |  | GTACTCTGTACAAGCACCTC |  |
| *PAX6-10F* | 10 | GTAGACACAGTGCTAACCTG | 243 |
| *PAX6-10R* |  | CCCGGAGCAAACAGGTTTAA |  |
| *PAX6-11F* | 11 | TTAAACCTGTTTGCTCCGGG | 208 |
| *PAX6-11R* |  | TTATGCAGGCCACCACCAGC |  |
| *PAX6-12F* | 12 | GCTGTGTGATGTGTTCCTCA | 300 |
| *PAX6-12R* |  | TGCAGCCTGCAGAAACAGTG |  |
| *PAX6-13F* | 13 | CATGTCTGTTTCTCAAAGGGA | 957 |
| *PAX6-13R* |  | GAACAATTAACTTTTGCTGGCC |  |
| *MIP-1F* | 1 | AAGGGGACTGTCCACCCAG | 298 |
| *MIP-1R* |  | GCCACCTGCAGAACATGCAG |  |
| *MIP-2F* | 2 | AGGAGGTAACACTGTGGCAG | 261 |
| *MIP-R\* |  | GAATCCTTGAATGAGAAGTTGC |  |
| *MIP-3F* | 3 | AAGCTGGGGTGCAGTAGGG | 247 |
| *MIP-3R* |  | GAGTGCTGGTACAGCAGCC |  |
| *MIP-4F* | 4 | CAGCGTTGCTGCTCTGTCC | 460 |
| *MIP-4R* |  | TGGGGAGGAAGGGAAGTTTG |  |
| *PITX3-1F* | 1 | CCCTGGTCTGCCATAAAGTG | 294 |
| *PITX3-1R* |  | TTTAGGGATTCCAAGGTCCA |  |
| *PITX3-2F* | 2 | GGCTGGGGTTGAGAAAGGCG | 339 |
| *PITX3-2R* |  | CCACTCGCTGGCTCCCACC |  |
| *PITX3-3F* | 3 | GCAGCCCCGGTGGGAGC | 400 |
| *PITX3-3R* |  | GGGAGGGGGCAGGTGGG |  |
| *PITX3-4F* | 4 | CCGTCTCTAGCCACCTCATC | 794 |
| *PITX3-4R* |  | CCAGTCAAAATGACCCCAGT |  |
| *HSF4-1F* | 1 | GGCAAACGCAGCACTTTC | 262 |
| *HSF4-1R* |  | GTTCACTGACGTGGAGGGAC |  |
| *HSF4 2-3F* | 2&3 | CGCTCACCCTCCTGGTC | 437 |
| *HSF4 2-3R* |  | AAGGCAGGCAGTCCCAG |  |
| *HSF4 4-5F* | 4&5 | GGGAATGAGCAAAGAGGAGG | 590 |
| *HSF4 4-5R* |  | GTGGAATGGGGTGTCGAG |  |
| *HSF4 6F* | 6 | TTCCTCCCTCACCTGGAAG | 205 |
| *HSF4 6R* |  | CTTGCAAGGGGACTTCTGG |  |
| *HSF4 7-8F* | 7&8 | GGAAGTGCAGGCCGAGG | 620 |
| *HSF4 7-8R* |  | CCCCTACAGCCATCTGGG |  |
| *HSF4 9F* | 9 | AGGGGTAGAGGGAGAAGTCAG | 355 |
| *HSF4 9R* |  | CTAGGAAGCTTTGTGGGCTG |  |
| *HSF4 10-11F* | 10&11 | TCTTGATGCATCTGGGTTCC | 405 |
| *HSF4 10-11R* |  | GACCAGAGGGCTTGACTCAG |  |
| *HSF4 12F* | 12 | TGTCACAGTGATTTCCCAGC | 204 |
| *HSF4 12R* |  | CAAGGTAGCTCAGCCCAATC |  |
| *HSF4 13F* | 13 | CTGAAGAAAGGAGGGGGAAC | 453 |
| *HSF4 13R* |  | CTGGACGCTTCTACAAATGC |  |
| *MAF-1F* | 1 | TGTGTGTGTGAGCGCGCTCGGA | 1381 |
| *MAF-1R* |  | AGCATGGCTCTAGAACTAGCAAGCC |  |
| *MAF-2F* | 2 | TACGCTGCGTTTGATCTTTG | 224 |
| *MAF-2R* |  | AGGTGGTTCTCCATGACTGC |  |
| *FTL 1-2F* | 1&2 | CCTATGTGCTCCGGATTGGTCA | 867 |
| *FTL 1-2R* |  | AGGGCTCACAAGACCGAACTCA |  |
| *FTL 3-4F* | 3&4 | GGACAGGGTGCGGAGAGTGATAAA | 783 |
| *TFL 3-4R* |  | ATCCCACCTCATCTTCCACACCA |  |
| *BFSP2-1F* | 1 | GTAAACCAAAAGCCACTGGACTCTG | 729 |
| *BFSP2-1R* |  | TGGGCTCCTAGTAAACCTCGGTAA |  |
| *BFSP2-2F* | 2 | CCCCA CCCTGACCAT TGTCCAAA | 478 |
| *BFSP2-2R* |  | AACCC TGCTC TAAACCAACT GAC |  |
| *BFSP2-3F* | 3 | TTTTGGGCTAC TCAGTTATGC TA | 276 |
| *BFSP2-3R* |  | CTTGTTATGAA GCACAGGCAGAC |  |
| *BFSP2-4F* | 4 | CAGTTGTGGA ATGAGAAAAG AAT | 377 |
| *BFSP2-4R* |  | CTAAACCGTTGT TATTTCTTCG TC |  |
| *BFSP2-5F* | 5 | GAAAGGCTGGGAAGGAAGGATGG | 366 |
| *BFSP2-5R* |  | AGTATCTGATGATCCTTGGGAGT |  |
| *BFSP2-6F* | 6 | TCCCACGGAGCCACTAAGAATAACC | 563 |
| *BFSP2-6R* |  | TGTATGTGAAT AGGAAAGGAAGG |  |
| *BFSP2-7F* | 7 | CCCAAGG TCTCACAGCA AATAAC | 247 |
| *BFSP2-7R* |  | CTCAAGCAACTTCTTGGGTCAGC |  |
